# Chronic syndesmotic instability – Current evidence on management

**DOI:** 10.1016/j.jcot.2024.102382

**Published:** 2024-02-23

**Authors:** Abdul-Rahman Gomaa, Lyndon Mason

**Affiliations:** a Human Anatomy and Resource Centre, University of Liverpool, Liverpool, UK; b Liverpool Orthopaedic and Trauma Service, Liverpool University Hospitals NHS Foundation Trust, Liverpool, UK

**Keywords:** Syndesmosis, Chronic syndesmotic instability, Syndesmotic reconstruction, Ligamentoplasty, Ankle instability

## Abstract

This review article discusses the current evidence on the management of chronic syndesmotic instability. Conservative treatment has a limited role, and surgical intervention is most commonly reported as the mainstay of treatment, however the literature consists of small case series and descriptions of operative techniques, and thus the evidence base for any treatment is weak. Surgical options include arthroscopic debridement alone, static fixation with cortical screws, dynamic fixation with suture-button devices, and ligamentous repair or augmentation.

## 1 Introduction

**Fig. 1:**
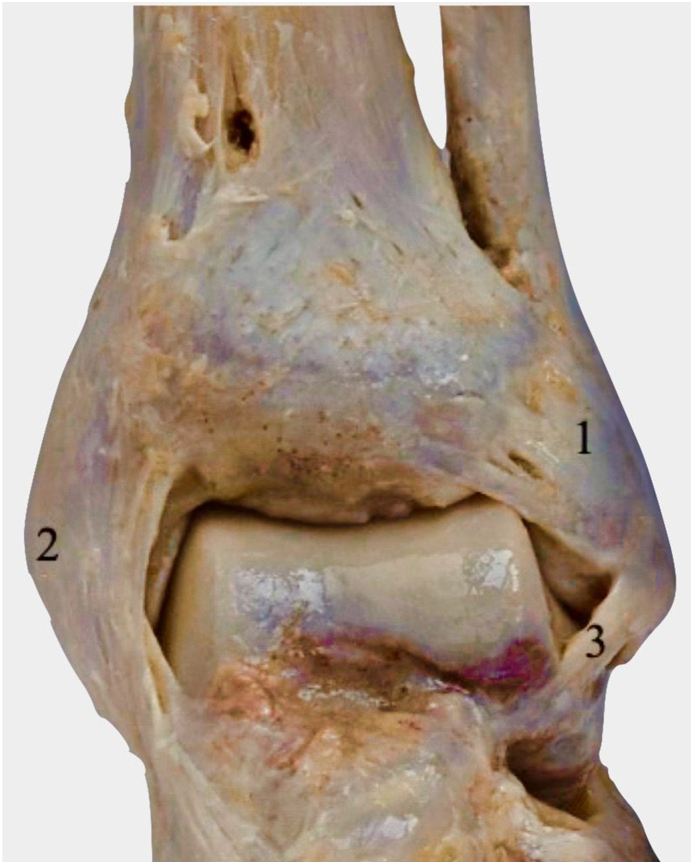

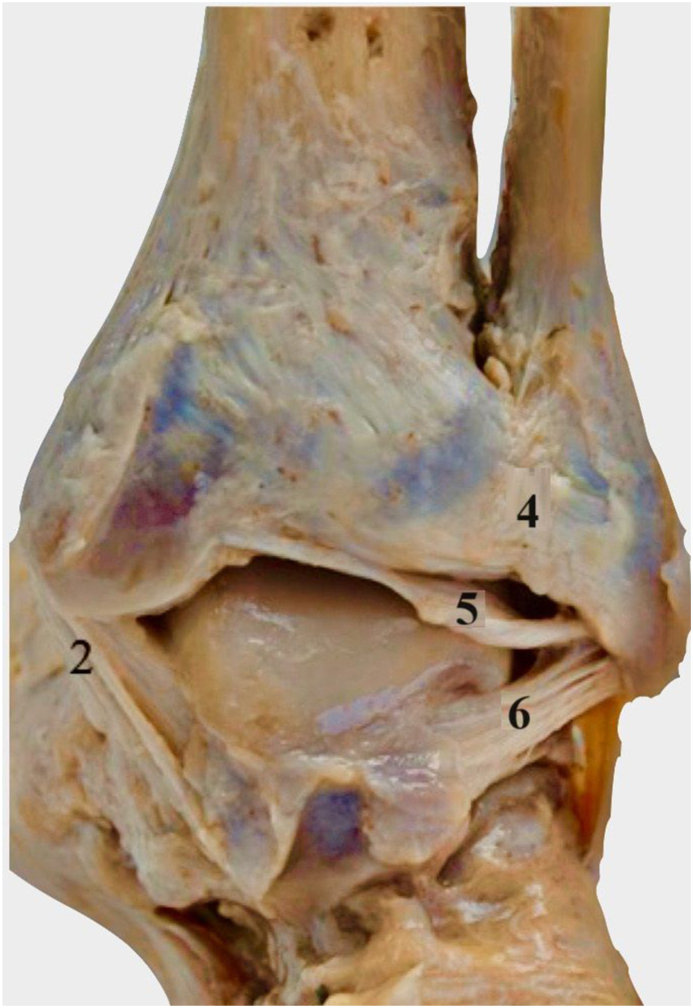
Cadaveric images demonstrating the ligaments of the ankle joint. A - Anterior view, B - Posterior view. 1 - Anterior inferior tibiofibular ligament, 2 - Deltoid (medial) ligament, 3 - Anterior talofibular ligament, 4 - Posterior inferior tibiofibular ligament, 5 – Intermalleolar ligament, 6 - Posterior talofibular ligament. IOL not shown.

Chronic syndesmotic instability (CSI) is the consequence of persistent ligamentous incompetence to any of the four syndesmotic ligaments (i.e. the anterior inferior tibiofibular ligament (AITFL), posterior inferior tibiofibular ligament (PITFL), interosseous ligament (IOL), and the interosseous membrane (IOM)) as shown in Fig. 1.^1^ Rotatory instability of the syndesmosis is primarily attributed to AITFL in external rotation, however internal rotation stability is not usually diminished until all ligaments are incompetent.^2^ Coronal stability of the syndesmosis (Y axis translation), or complete syndesmosis diastasis, is usually attributed to the posterior syndesmosis, contributing 40–45% of the resistance, with the AITFL accounting for approximately 35%.^3^ Significant injuries to two syndesmosis components result in a loss exceeding 50% of diastasis resistance, potentially leading to instability. Massri Pugin et al. concluded that under arthroscopic evaluation, AITFL, PITFL and IOL needed to be incompetent to produce coronal instability.^4^ Sagittal instability of the syndesmosis is more common than coronal instability, with also much greater displacement reported in the sagittal plane than in the coronal plane.^5^ Secondly, as described by Lambert et al., the syndesmotic ligaments are also responsible for talocrural stability, in coronal stability (Y axis translation) and Z axis rotation.^6^

Injuries to the syndesmosis commonly coexist with ankle fractures in up to 20% of cases and ankle sprains in up to 25% of athletes.^7^, ^8^, ^9^, ^10^, ^11^ If missed or neglected, CSI can lead to persistent pain and dysfunction due to misalignment and instability at the talocrural joint; leading to increased morbidity, pain, stiffness, instability, degenerative osteoarthritic changes, heterotopic ossification and poorer clinical outcomes.^12^, ^13^, ^14^, ^15^, ^16^, ^17^, ^18^

The literature lacks consensus regarding the precise time frame that defines the CSI. Different authors have defined chronic instability as occurring anywhere between six weeks to six months after the initial injury.^12^^,^^19^, ^20^, ^21^, ^22^, ^23^, ^24^ Several risk factors have been identified that could predispose patients to developing CSI, including: diabetes with associated neuropathy, obesity and ankle fractures with posterior malleolar fragments.^25^, ^26^, ^27^ It is important to closely monitor patients with these risk factors for the development of CSI. However, there is a sparsity of evidence on how patients with these risk factors should be managed. Accurate reduction and more stable fixation should be considered for patients suffering with obesity or a posterior malleolar fracture whilst a stronger mechanical construct or a more conservative postoperative protocol have been suggested as further areas of research for neuropathic patients.^25^, ^26^, ^27^ The aim of this review article is to summarise the current evidence on management of CSI.

## 2 Conservative management

A recent European consensus statement noted that conservative treatment outcomes for CSI are generally regarded as unfavourable.^21^ However, it acknowledges recent advances in neuromuscular training functional instability treatment with promising initial results, leading to the development of further research on the outcomes of intensive rehabilitation within the context of chronic ankle instability.^28^, ^29^, ^30^, ^31^ Nevertheless, surgeons continue to reserve non-operative treatment as a preventive measure to reduce recurrences following surgery and to enhance overall outcomes.

The consensus statement from the Asia-Pacific Knee, Arthroscopy, and Sports Medicine Society (APKASS) recommends nonoperative treatment for syndesmotic injuries without an ankle fracture, specifically targeting symptomatic sprains without diastasis and no signs of joint instability on imaging. This management approach received unanimous agreement (100% vote) from the voters. However, it was strongly agreed upon (75% agreement) that syndesmotic injuries treated nonoperatively without a fracture generally take a longer time to heal compared to cases where surgical interventions were employed.^32^

## 3 Surgical management

**Fig. 2:**
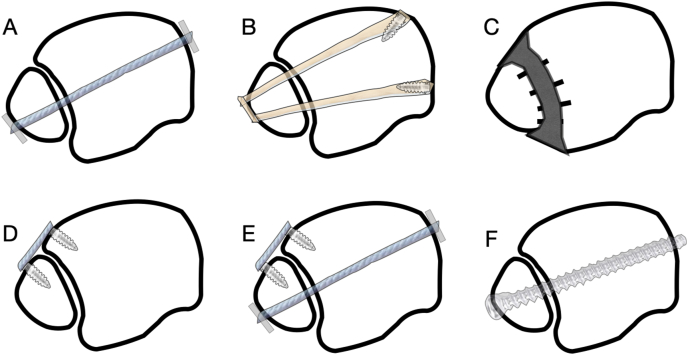
Examples of different CSI management. A – Dynamic fixation, B – Tendon reconstruction, C – Fusion, D – AITFL reconstruction, E − Combined AITFL reconstruction and dynamic fixation, F – Static fixation.

The mainstay of surgical treatment of CSI can be split into arthroscopic debridement coupled with either static fixation using one or more cortical screws or dynamic fixation using suture‐button devices as demonstrated in Fig. 2. Although high-quality evidence is limited concerning the surgical treatment of CSI, the available studies consistently demonstrate favourable outcomes for the different surgical options.^33^^,^^34^

In acute syndesmotic injury, dynamic fixation of the syndesmosis using dynamic devices was found to yield superior outcomes when compared to static fixation using screw in both cadaveric studies as well as clinical trials.^35^, ^36^, ^37^, ^38^, ^39^ In a recent meta-analysis comparing fixation methods biomechanically, screw fixation displayed greater strength, yet load, torque and rotation at failure were comparable to suture-button devices. It was theorised that suture-button devices exhibited slight micro-motion, mimicking natural mobility that can be advantageous for ligament healing.^40^ A meta-analysis of randomised control trials comparing dynamic stabilisation of syndesmosis injuries against screw fixation noted that dynamic fixation showed promising results in reducing complications and improving clinical outcomes compared to static screw fixation, particularly in cases of malreduction and clinical instability or diastasis, even at a follow-up of 2 years.^36^ Moreover, dynamic fixation was associated with a lower risk of reoperation when compared to static fixation utilising permanent screws. However, it is worth noting that these studies did not solely focus on CSI.

Fixation of the syndesmosis in CSI can be further augmented with suture repair of damaged syndesmotic ligaments or grafting using different tendons such as: peroneus brevis,^41^ peroneus longus,^42^^,^^43^ semitendinosus,^44^, ^45^, ^46^, ^47^, ^48^ a free gracilis autograft,^49^, ^50^, ^51^, ^52^, ^53^ periosteal flap,^54^ and a plantaris autograft.^54^ Arthrodesis of the syndesmosis is another surgical option which can be considered for certain cases whereby there is arthrosis or as a salvage procedure.

### 3.1 Arthroscopic debridement alone

Arthroscopy is commonly used as a diagnostic aid in CSI and utilised for debriding the ankle joint, although few studies have examined the role of arthroscopic debridement alone for the treatment of CSI. In the two published series by Ogilvie-Harris et al. they noted that arthroscopic debridement of the syndesmosis in CSI significantly reduces pain and improves activity level.^55^^,^^56^ Han et al. is the only reported study to compare arthroscopic debridement alone to arthroscopic debridement combined with screw fixation for CSI and found no statistical difference in outcome between interventions.^57^

### 3.2 Static fixation

Static fixation of the syndesmosis for CSI is achieved using one or more cortical screws. There remains an ongoing debate surrounding the optimal number screws number of cortices, location, diameter, material, and positioning. In managing CSI, multiple studies described and examined the role of standalone cortical screws as a method of static fixation.^57^, ^58^, ^59^, ^60^, ^61^, ^62^, ^63^, ^64^ They ranged from descriptions of operative techniques to small cases series which utilised a small number of participants that ranged from 1 to 12 participants per study. There was significant heterogeneity within the nature of static fixation used. The number of screws ranged from 1 to 3 screws. The size of the screw used ranged from 4.0 mm to 7.3 mm with some studies not reporting the size of screw they used. Finally, whilst most studies described a fixation which engaged all 4 cortices, one study described engaging 3 cortices^61^ whilst another one did not specify how many cortices the screw engaged.^57^

In general, these studies reported good patient reported outcome measures (PROMs) and functional outcome improvements, including the ability to restore tibiofibular kinematics as well as satisfactory radiographic assessment.^58^^,^^60^^,^^62^^,^^64^ Han et al. was the only study to directly compare the outcomes of arthroscopic debridement alone to arthroscopic debridement combined with screw fixation.^57^ They reported no significant statistical difference between the two interventions. Based on their findings, the authors concluded that arthroscopic debridement alone could yield similar outcomes in cases of CSI without combined medial ankle instability and lateral talus displacement.^57^

### 3.3 Dynamic fixation

Regarding CSI, Ryan et al. reported outcomes of using standalone suture-button fixation for the syndesmosis in a series of 19 patients with CSI.^65^ The syndesmosis was debrided and stabilised using 2 suture-buttons in 13 patients and 3 suture-buttons in 6 patients in combination with arthroscopic examination and debridement. In their series, they noted significant improvements in PROMs and radiographic measurements, with 86% of patients able to return to running and 79% able to return to their preinjury level of sport.

More recently, Kingston et al. conducted a comparison between distal tibiofibular arthrodesis (32 ankles) and delayed debridement, reduction, and syndesmosis stabilisation with suture endobuttons (39 ankles) for cases of CSI.^66^ Despite improvements observed in both groups, there was no statistically significant difference in postoperative PROMs and radiographic measurements between the two groups at follow up. However, the arthrodesis group had a higher reoperation rate, primarily due to hardware removal (18.8% vs 2.6%).

### 3.4 Combined dynamic and static fixation

For high-level athletes and individuals with high physical demands, a combination of dynamic and static fixation has been recommended to protect the syndesmotic repair while facilitating rapid restoration of the biomechanical action of the syndesmosis.^67^, ^68^, ^69^, ^70^ In a series of 32 patients by Colcuc et al. three ligamentous augmentation techniques (suture of the anterior inferior tibiofibular ligament (AITFL), ligament repair using periosteal flaps, and autogenous plantaris tendon graft) were performed in combination with static fixation using a 3.5 mm tricortical transfixation screw and a dynamic fixation using a suture-button device.^54^ Significant improvements in PROM scores were observed in all three groups compared to their preoperative levels. Whilst there were no complications following the arthroscopies and reconstructive surgeries, in two patients, removal of the suture-button device was necessary due to symptomatic granuloma formation.

Stake et al. published their series of 11 patients with CSI that was managed by either open (5 patients) or arthroscopic (6 patients) debridement of the syndesmosis and fixed with a combination of a suture-button and either a 3.5 mm or 4.5 mm quadcortical screw.^71^ They reported significant improvements in PROMs and radiological assessments, comparable with other published series. Nevertheless, they encountered five complications, with three cases of hardware-related pain, and most patients exhibited radiographic indications of progressing osteoarthritis. Of the five complications, three (3/5, 60%) were associated with open debridement while two (2/6, 33.3%) were associated with arthroscopic debridement.

### 3.5 Ligamentous reconstruction

Many authors have attempted to use various ligamentous grafts concurrently to augment syndesmotic fixation or in isolation to reconstruct the different syndesmotic ligaments as outlined in Table 1. It was theorised that reconstructing the ligaments would act as a way of restoring the anatomical and biomechanical functions of the syndesmosis.^42^^,^^47^^,^^52^^,^^72^ In a recent systematic review of five studies conducted by Xu et al., the reconstruction of CSI using an autogenous (peroneus longus, semitendinosus, gracilis or plantaris) tendon graft for the distal tibiofibular syndesmosis showed a favourable therapeutic outcome, as evident from improved subjective symptoms and objective evaluation scores.^73^ The review suggested that the IOL could be a suitable reconstruction target in the management of chronic syndesmosis injury. Since then, further studies and operative techniques have been reported that describe ligament-only reconstructive techniques.

**Table 1:** A summary of the reported literature on the different ligamentous repair techniques described for CSI. n.r. - Not reported.

Author	Year	Publication type	Number	Methods	Syndesmotic fixation details	Ligamentous augmentation	Reconstructed ligament(s)	Key findings
Beumer et al.^59^	2000	Case series	9	AITFL reconstruction using a bone block advancement and screw fixation	1x quadcortical screw (unspecified size)	N/A - AITFL reconstruction using a bone block advancement	AITFL	The results given are encouraging
Castaing et al.^41^	1961	Operative technique	n.r.	Open lateral ankle ligamentoplasty	None	Peroneus brevis tendon	n.r.	Described a novel method for the surgical reconstruction of the syndesmotic ligaments
Colcuc et al.^54^	2016	Case series	32	Arthroscopic examination + open reconstructive surgery (suture of the anterior inferior tibiofibular ligament (n = 10), ligament repair using periosteal flaps (n = 12), or autogenous plantaris tendon graft (n = 10)) and syndesmotic fixation screw and a suture-button.	1x screw (unspecified size and number of cortices) and 1x suture-button	Periosteal flaps or autogenous plantaris tendon	n.r.	Significant improvements in PROMs postoperatively. Two patients required suture-button removal due to granuloma formation, no other complications were reported.
Connors et al.^82^	2020	Operative technique	1	Open debridement, reduction of the syndesmosis followed by bone tunnels, syndesmotic reduction with screws and reconstruction using graft.	2x quadcortical screw (unspecified size)	Split semitendinosus allograft	AITFL + IOL	Described a novel method for the surgical reconstruction of the AITFL and interosseus ligaments
Dekker et al.^44^	2017	Case series	6	Debridement of the syndesmosis and fixation using a suture-button and a double limb semitendinosus allograft reconstruction and a deltoid ligament repair	1x suture-button	Semitendinosus tendon	IOL	Early outcomes have been excellent. One patient experienced an early postoperative fibula fracture, which necessitated interventions including open reduction, internal fixation of the fibula, and revision of medial malleolar fixation.
Grass et al.^42^	2003	Case series	16	Syndesmotic fixation using a screw, bone tunnels and ligament reconstruction	1 × 3.5 mm tricortical screw	Peroneus longus tendon	3 syndesmotic ligaments (PITFL, IOL and AITFL)	15 out of 16 patients expressed pain alleviation and indicated their willingness to undergo the procedure again at an average follow-up of approximately 16 months.
Lui et al.^43^	2010	Operative technique	n.r.	Arthroscopy, debridement, bone tunnel and 3 ligament reconstruction	1x tricortical screw (unspecified size)	Peroneus longus tendon	3 syndesmotic ligaments	Described a novel method for the surgical reconstruction of the distal tibiofibular syndesmosis
Mansur et al.^45^	2021	Case report	1	Medial space debridement. Anatomical realignment of the distal fibula with a lengthening derotational osteotomy and tibiofibular syndesmosis reconstruction using graft	1x screw (unspecified size and number of cortices)	Autologous semitendinosus tendon	AITFL	A semitendinosus tendon graft for syndesmosis reconstruction along with fibular lengthening and realignment leads to improved ankle stability, function and positive outcomes at the one-year mark.
Michelitsch et al.^49^	2014	Case report	1	Open debridement, osteotomy, syndesmotic stabilisation with k-wire stabilisation, bone tunnels, tendon placement and syndesmotic fixation using screw	1x quadcortical screw (unspecified size)	Gracilis tendon	n.r.	Anatomical conditions can be reconstructed exactly in CSI using a lengthening/derotational osteotomy and reconstruction of syndesmosis using a gracilis tendon
Moravek et al.^46^	2010	Case series	6	As per bespoke treatment algorithm	1x suture-button	Semitendinosus allograft	IOL	The algorithmic approach coupled with novel allograft ligament reconstruction offers an effective solution for managing challenging syndesmosis problems. Six consecutive patients were treated using this approach, resulting in positive outcomes, with improved ankle stability and function. While one patient experienced an early postoperative fibular fracture, the majority of patients were satisfied with the procedure's results.
Morris et al.^47^	2009	Case series	8	Debridement, reduction of the syndesmosis then reconstruction using a graft	1 × 4.5 mm quadcortical screw	Semitendinosus autograft	IOL and the AITFL	Patients reported a significant improvements in PROMs. However, one patient necessitated arthrodesis due to preexisting degenerative disease during reconstruction.
Nelson et al.^76^	2006	Case series	50	Open reduction, internal fixation of ankle fracture followed by either AITFL repair (graft n: 23) or suture (n:14)	None	Extensor digitorum longus (n: 23)	AITFL	Repairing the AITFL anatomically restores ankle stability and bone repair, facilitating swift return to activities. Syndesmotic screw fixation wasn't required for transmalleolar ankle fractures when using these repair techniques.
Olory et al.^50^	2023	Operative technique	n.r.	Debridement and reduction of the syndesmosis followed by reconstruction of syndesmosis using graft and deltoid ligament reconstruction	1x screw (unspecified size and number of cortices) or 1x suture-button	Autogenous gracilis tendon	IOL and the AITFL	Described a novel method for reconstruction of the syndesmosis AITFL and IOL and the concomitant reconstruction of the anterior bundle of the DL by implementing a single and continuous gracilis autograft.
Sharafeldin et al.^51^	2023	Case report	1	Complete debridement of fibrotic tissue was done, followed by an open arthrotomy, and debridement of the fibrotic issue was achieved under direct vision	None	Autogenous gracilis tendon	Entire syndesmosis	Significant improvements in PROMs and function.
Vilá-Rico et al.^52^	2018	Operative technique	n.r.	Arthroscopy, debridement of the syndesmosis and fixation using suture-button followed by AITFL reconstruction	1-2x suture-button	Gracilis or extensor hallucis longus allograft	AITFL	Described a novel method for arthroscopic reconstruction of the AITFL supplemented by percutaneous syndesmotic suture-button constructs for cases of chronic syndesmotic instability.
Wagener et al.^64^	2011	Case series	12	Arthroscopic examination, debridement and AITFL reconstruction using a bone block advancement and screw fixation	1x quadcortical screw (unspecified size)	N/A - AITFL reconstruction using a bone block advancement	AITFL	Anatomic reconstruction of the anterior syndesmosis leads to favourable outcomes, with the significant improvement in PROMs postoperatively.
Yasui et al.^53^	2011	Case series	6	Arthroscopic examination, debridement of syndesmosis, AITFL reconstruction using graft and fixation of syndesmosis using screw	1 × 3.5 mm tricortical screw	Autogenous gracilis tendon	AITFL	Significant improvement in PROMs following reconstruction at final follow up.
Zamzami et al.^48^	2009	Case series	11	Arthroscopy and debridement of syndesmosis followed by open ligament reconstruction±gastrocnemius release	1 × 3.5 mm tricortical screw	Semitendinosus tendon	AITFL + PITFL	The technique presented yields outstanding results, with no arthroscopic surgery-related complications noted. Two instances exhibited superficial stitch abscesses at the reconstruction sites, both of which resolved with local wound care and no lasting issues.

Several ligament-only reconstructive techniques have been described independently, but they have not been adequately studied. Castaing et al. introduced a lateral ankle ligamentoplasty technique using the peroneus brevis tendon as a modification of the Watson–Jones technique for CSI.^41^^,^^74^ Subsequently in 1975, Castaing et al. published their medium-term follow-up results for 22 patients, noting that 19 patients (86.3%) achieved good outcomes, while 3 patients (13.6%) had fair results with a slight deterioration observed after 8 years of follow-up.^75^

Nelson described a technique for reconstructing the AITFL using the extensor digitorum longus tendon as a graft in 23 out of 50 patients with acute ankle fractures and associated syndesmotic injuries.^76^ However, he did not provide any follow-up outcome data or compare this group to the other group that underwent non-absorbable suture repair. In a more recent case report, Sharafeldin et al. described a technique where they reconstructed the entire syndesmosis using a single gracilis tendon autograft.^51^ The patient showed significant improvements in symptoms, function, and various patient-reported outcome measures (PROMs) at the 3-year follow-up.

### 3.6 Ligamentous augmentation of syndesmotic fixation

There is significant heterogeneity within the literature regarding this combined technique as outlined in Table 1. Various ligamentous augmentation techniques have been used for syndesmotic fixation, including the use of grafts such as the extensor hallucis longus,^52^ gracilis,^49^^,^^50^^,^^52^^,^^53^ peroneus longus,^42^^,^^43^ and semitendinosus tendons.^44^, ^45^, ^46^, ^47^, ^48^ Most of the described techniques focus on reconstructing either the AITFL^45^^,^^52^^,^^53^^,^^59^^,^^64^ or the IOL,^44^^,^^46^ while some methods address the simultaneous reconstruction of two^47^^,^^48^^,^^50^ or three^42^^,^^43^ syndesmotic ligaments. These ligament reconstructions are typically combined with either one syndesmotic screw fixation or one to two suture-buttons. The existing literature mainly consists of small retrospective case series and descriptions of operative techniques, which limits the evidence to weak and low-level sources.

### 3.7 Arthrodesis

Arthrodesis of the distal tibiofibular joint may be considered as a viable salvage procedure in situations where CSI is accompanied by arthrosis or has not responded to previous surgical management as outlined by Peña and Coetzee.^77^ Olson et al. were the first to report on arthrodesis for CSI in a small series of ten patients.^78^ Their findings suggested that CSI following ankle fractures could be successfully salvaged by performing reduction and arthrodesis of the distal tibiofibular articulation. Consequently, Kingston et al. conducted a comparison between delayed debridement, reduction, and stabilisation using suture endobuttons and arthrodesis for CSI.^66^ Their findings revealed no significant differences postoperatively between the stabilisation and arthrodesis groups regarding Kellgren scores, medial clear space, talocrural angle, and talar tilt. Both groups demonstrated uniform improvement in clinical outcomes; however, the arthrodesis cohort exhibited a higher reoperation rate, particularly due to hardware-related complications.

Sun et al. reported on their series of 8 patients which underwent arthrodesis for CSI using plate and screw fixation.^79^ Three months postoperatively, all patients were able to bear full weight and expressed satisfaction with the results, with seven of them returning to sports activities. There were significant improvements in PROMs during the final follow-up. However, four patients experienced mild restrictions in ankle range of motion compared to the unaffected side.

In a narrative review conducted by Lim et al., a comparison of functional outcomes between arthrodesis and ligament reconstruction in patients with syndesmotic injuries revealed similar positive results for both interventions, without any significant differences observed between them.^80^

## 4 Controversies

Numerous debates revolve around the diagnosis and management of CSI. While arthroscopy and clinical evaluation are generally accepted as the gold standard diagnostic methods, agreement is lacking regarding the precise arthroscopic diagnostic approach and the radiographic criteria to be evaluated through plain radiographs, CT scans, or MRIs.^21^^,^^24^^,^^69^ Moreover, there is considerable disparity in defining the duration that classifies syndesmotic injuries as chronic, ranging from 6 weeks to 6 months.^12^^,^^19^^,^^20^^,^^24^^,^^54^^,^^81^ Lastly, although numerous surgical techniques and case series have been documented in literature with broadly satisfactory outcomes, they all exhibit noteworthy constraints, which restrict their broader applicability.

## 5 Conclusion

The management of CSI continues to be a subject of controversy, given the array of surgical treatment choices which include arthroscopic debridement alone, static fixation with cortical screws, dynamic fixation with suture-button devices, and ligamentous repair or augmentation and yield comparably positive outcomes. Clarifying the definition of CSI is important in order to ensure consistent timely diagnosis and standardisation of treatment. We propose that syndesmotic instability lasting more than 6 weeks should be classed as CSI to facilitate prompt management. To enhance the evidence foundation and research quality in CSI management, comprehensive prospective randomised trials would be beneficial for identifying the techniques with most clinical gain.

## Ethics statement

Institutional review board approval was not required for this literature review.

## Funding

This research did not receive any specific grant from funding agencies in the public, commercial or not-for-profit sectors.

## CRediT authorship contribution statement

**Abdul-Rahman Gomaa:** Conceptualization, Methodology, Investigation, Writing – original draft, Project administration. **Lyndon Mason:** Conceptualization, Methodology, Writing – review & editing, Supervision.

## Declaration of competing interest

The authors declare that they have no known competing financial interests or personal relationships that could have appeared to influence the work reported in this paper.
